# Cane molasses as a source of precursors in the bioproduction of tryptophan by *Bacillus subtilis*

**Published:** 2013-09

**Authors:** Marzieh Dehghan Shasaltaneh, Zahra Moosavi-Nejad, Sara Gharavi, Jamshid Fooladi

**Affiliations:** Department of Biology, Faculty of Basic Sciences, Alzahra University, Tehran, Iran

**Keywords:** Tryptophan, *Bacillus subtilis*, cane molasses, indole, tryptophan synthase

## Abstract

**Background and Objectives:**

The essential amino acid L-tryptophan can be produced by a condensation reaction between indole and L-serine, catalyzed by *B. subtilis* with tryptophan synthase activity. Application of the tryptophan is widespread in the biotechnology domain and is sometimes added to feed products as a food fortifier.

**Materials and Methods:**

The optimum concentration of the Iranian cane molasses was determined by measuring the amount of biomass after growth in 1 to 30 g/mL of molasses. The maximum amount of biomass was obtained in 10 g/mL molasses. Chromatographic methods, TLC and HPLC, were used to assay the amount of tryptophan produced in the presence of precursors of tryptophan production (indole and serine) and/or molasses.

**Results:**

Our results indicate the importance of the Iranian cane molasses not only as carbon source, but also as a source of precursors for tryptophan production.

**Conclusion:**

This report evaluates the potential of cane molasses as an economical source for tryptophan production by *B. subtilis*, hence eliminating the requirement for additional serine and indole as precursors.

## INTRODUCTION

**Fig. 1:**
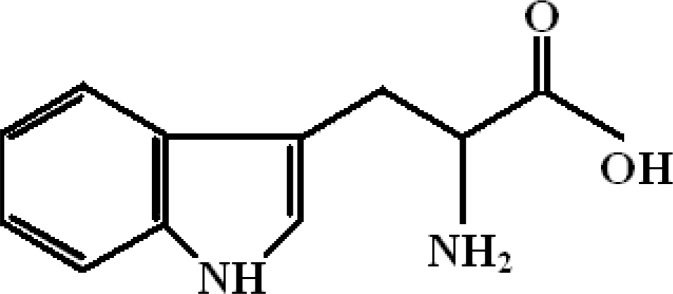
The structure of L-tryptophan.

The chemical nomenclature of tryptophan (Trp) is α-amino-β-indylpropionic acid (Fig. 1). L-tryptophan is one of essential amino acids in human and animals which participates in protein synthesis and metabolic regulation and is widespread in nature. For medical applications, L-tryptophan can be converted to hormones, such as 5-hydroxy tryptophan which has a regulatory function in the central nervous system (1) or converted into physiological active substances such as alkaloids (2) and coenzymes through metabolism (3). Thus L-tryptophan plays an important role as an antidepressant (4) and anti-anxiety agents (5), eliminates nervous tension (6), improves sleep (7) and has potential use in preventing or treating pellagra (8).

In the food industry, L-tryptophan can be used as food additives to strengthen the protein utilization of body (9). Moreover, L-tryptophan is resistant to oxidation and hence can function to prevent the decomposition of fish and powder milk (10).

Currently, the industrial production of L-tryptophan is mostly for feed and pharmaceutical purposes. The first reports on L-tryptophan production date back to the beginning of the 20th century (11). To meet the increasing demand for this amino acid, a wide variety of chemical and biotechnological methods have since been developed. Chemical synthesis was the first method used in an industrial scale process (12). By the end of the 80s, production of L-tryptophan was carried out through chemical, enzymatic and fermentation processes (13).

The enzymatic production of L-tryptophan from precursors involves a single reaction step, catalyzed by enzymes, either tryptophan synthase (TSase; EC 4.2.1.20) or tryptophanase (TPase; EC 4.1.99.1) or by a variety of microorganisms with these enzyme activities (14). Tryptophan synthetase catalyzes the final step in the biosynthesis of tryptophan, the convertion of indole 3-glycerol phosphate to tryptophan (15) and hence many attempts have been made to overproduce this important amino acid in a number of bacteria including *Escherichia coli* (16), *Bacillus subtilis* (17), *Brevibacterium flavum* (18) and *Pseudomonas aeruginosa* (19). Biotransformation uses *E. coli* cells, which overproduce tryptophan synthase, indole and L-serine as substrates. The majority of tryptophan production, however, is by microbial fermentation mainly with *Corynebacterium* and *E. coli* (20). Microbial fermentation strains allow the production of various amino acids from cheap and renewable carbon sources such as sucrose, glucose or molasses and are therefore, usually more favorable than biotransformation processes (21).

In this study, the potentials of *Bacillus subtilis* with tryptophan synthase activity was evaluated using cane molasses as a rich carbon source as well as serine and indole as precursors in the enzymatic reaction carried out by the bacteria.

## MATERIALS AND METHODS

### Chemicals

L-tryptophan, L-serine and indole were purchased from MERCK (Germany) and cane molasses, a by-product from sugar production was purchased from a sugar factory (Karaj, Iran). Percentage of dry mass (oBx), percentage of sugar (Pol), the percentage of sugar purity (Q) and pH were measured for Iranian cane molasses (Table 1) (22).

**Table 1:** Iranian cane molasses composition (22).

°Bx (%)^a^	73.4
Pol (%)^b^	38.6
Q (%)^c^	52
pH	5.1

a Total soluble solids (w/w)

b Sucrose content (g/100 mL cane molasses)

c Purity percentage = (Pol/oBx) × 100

### Microorganism

The microorganism used is *B. subtilis* (ATCC 6633) from Iranian Research Organization for Science and Technology (IROST) which is capable of producing tryptophan synthase enzyme.

### Preparation of standard stock solutions

Standard stock solutions of tryptophan (0.001 M) was prepared in 10% (V/V) *n*-butanol and stored at 5°C as stock solutions for set up every 3 weeks (23) and working solution was prepared fresh for every experiment. Standard stock solution of serine (0.001 M) was prepared in water. Ninhydrin solution (0.1 g/100) was prepared in 100 mL acetone (24).

### Protein analysis

Protein production was determined by the Bradford method, with bovine serum albumin as standard. One milliliter of culture medium was used to assay the protein every hour (25). This assay is very rapid; consequently, the dye binding process was completed in approximately 2 min with color stability for 1 h.

### Sugar analysis

Total sugar was determined by the phenol sulfuric acid method, with sucrose sugar as a standard. One milliliter of culture medium was used to assay the sugar every hour (26).

### Instrumentations

Spectrophotometer (CECIL 9000, England) was used for protein and sugar assay. Total soluble solids were measured with HAENSCH refractometer (SCHMIDT, Germany) and HAENSCH polarimeter (SCHMIDT, Germany) was used for the determination of the amount of sucrose in cane molasses. The HPLC system consists of water liquid chromatography (Millford, MA, USA) equipped with a 600E multisolvent delivery system, an in-line degasser, a manual injection with 20 µL loop (Rheodyne 7125). Empower software was used for controlling the analytical system and data processing. CAMAG TLC scanner II was used to scan the TLC. CATS 3 software was used for analyzing the data.

### Culture medium

Lyophilized cells of *B. subtilis* strain were grown on nutrient agar slant at 4°C. Inoculums were prepared by the addition of one loopful of culture into 100 mL broth medium containing 0.5 g each of yeast extract and NaCl, 1 g each of peptone and glucose, 0.5g ammonium sulfate (pre-culture 1) and incubated at 28°C for 6 h on a rotary shaker at 150 rpm. Subsequently, the cells of pre-culture 1 were transferred to the same medium and were also incubated under the same conditions (pre-culture 2).

The actual determination of tryptophan production was carried out by inoculating 1 mL of the cells of pre-culture 2 into 100 mL solution in Erlenmeyer flasks containing 1 g peptone, 0.5 g yeast extract, 0.5 g NaCl, 0.05 g ammonium sulfate with the following supplements: 10 g/mL cane molasses (sample 1), 1 g/100 sucrose (sample 2), 0.1 g/100 indole and 1 g/100 sucrose (sample 3), 0.1 g/100 indole and 10 g/mL cane molasses (sample 4), 0.1 g/100 indole, 10 g/mL cane molasses and 0.2 g/100 serine (sample 5), 0.1 g/100 indole, 1 g/100 sucrose and 0.2 g/100 serine (sample 6).

In order to prevent Maillard reaction from taking place, cane molasses were sterilized separately at 121°C for 15 min or passed through a 0.22 µ Millipore filter. After centrifugation of the cells (20 min in 14000 g, 4 °C) (27), the supernatants were prepared for TLC and scan TLC.

In order to investigate the need for carbon source in tryptophan production, 1 mL of pre culture 2 was inoculated into the above medium containing just 10 g/mL cane molasses and their additives. Cane molasses concentrations were measured as grams per one liter of medium (W/V and not V/V) because of its high viscosity and thus to avoid error in volume measurement and transfer. Subsequently, the cell suspension was centrifuged and the biomass washed with a 0.9 g/100 NaCl solution and re-centrifuged (20). Next, the supernatant was removed. All further assessments of tryptophan synthesis were carried out using the supernatant of resting cells.

### Measurements of cell growth

Growth in the test medium was evaluated by measuring OD_620_ in a spectrophotometer every hour.

### Dry cell concentration determination

Samples used for growth curve were centrifuged (14000 g) for 20 min every hour. The pellet was suspended in distilled water and re-centrifuged. Biomass was determined by weighing after drying at 65°C for 24 h.

### HPLC method

The liquid chromatographic method was used for the determination of tryptophan with UV-Vis detector at 220 nm. Separation was carried out using C18 columns of 250 mm × 4.6 mm (Spherical, Optimal ODS-H, Capital HPLC, UK). The mobile phase employed was a mixture of solution A (5.6 mL triethylamine, TEA, adjusted to pH = 3 with H_3_PO_4_; the total volume of the mixture was then increased to 500 mL with H_2_O) and solution B (acetonitrile) (80:20). A volume of 20 µL was injected into the column for quantitative analysis and the mobile phase was allowed to run for 60 min with the temperature of the analytical column constant at 25 °C. The calibration curve and quantitative evaluations were carried out at 220 nm. The result was obtained by comparing the chromatographic peak area with that of the external standard. The results were compared to the area under the standard peak (0.001 M) obtained from the calibration graph (28).

### DTLC method

The amino acid produced was identified by thin-layer chromatography on silica gel 60 F_254_ plates (MERCK) with a solvent system of *n*-butanol/acetic acid/water (65:13:22 V/V/V) (29). Briefly, the supernatant from centrifugation of culture medium was mixed with *n*-butanol solvent (1:2) and the mixture vigorously vortexed and then incubated at room temperature for 3 h so that two phases were formed after separation of the organic phase. Following evaporation, 50 µL of the organic phase was loaded for TLC with the help of Hamilton syringe (30). The chromatogram was developed in rectangular glass chamber pre-equilibrated with the solution system for 15 min after which the chromatogram was dried at 60 °C for 10 min. A solution of the ninhydrin reagent was sprayed on the TLC which was subsequently dried again for 5 min at 110 °C. The identification was confirmed by R_f_ obtained by the comparison of tryptophan standard and sample.

### TLC scanning method

To measure tryptophan concentration in each sample, TLC scanning method was performed in the wavelength of 254 nm (LINOMAT IV model) (31). The scanning results were analyzed using CATS 3 software to calculate peak areas.

### Statistical Analysis

All experiments were carried out in triplicate, and the mean, standard error and P-values were calculated using SPSS (version 16.0) Software.

## RESULTS

### Determination of tryptophan concentration in medium

Due to the interference of tryptophan present in yeast extract and peptone, the culture medium was checked for tryptophan (0.5% in yeast extract and 1%, in peptone) using TLC and the subsequent scanning. The concentration of tryptophan was measured as 0.22 ± 0.05 mM and 0.14 ± 0.03 mM for yeast extract and peptone, respectively.

### Determination of optimum cane molasses concentration for cell growth

**Fig. 2:**
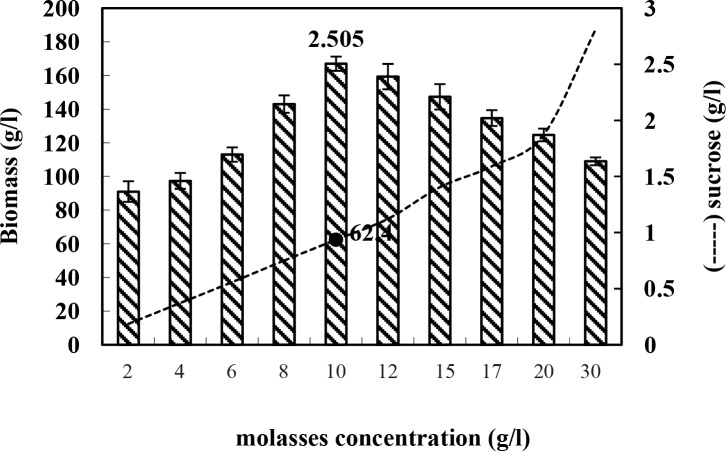
Determination of optimum cane molasses concentration for *B. subtilis.*

In order to determine the optimum cane molasses concentration for cell growth, the amount of bacterial biomass was measured in different concentrations of cane molasses (Fig. 2), expressed by percentage of biomass weight per volume (% W/V) due to its high viscosity. In each concentration of molasses in the medium, cell suspension of stationary phase was centrifuged and weighed. The concentrations of molasses corresponding to the highest biomass amount were selected as optimum cane molasses concentration for bacterial growth. The results are mean values of triplicate readings. As seen in Fig. 2, the maximum amount of biomass was obtained using 10 g/mL cane molasses concentration. Henceforth, the following experiments were performed using 10 g/mL cane molasses concentration.

### Investigation of *B. subtilis* metabolism

**Fig. 3:**
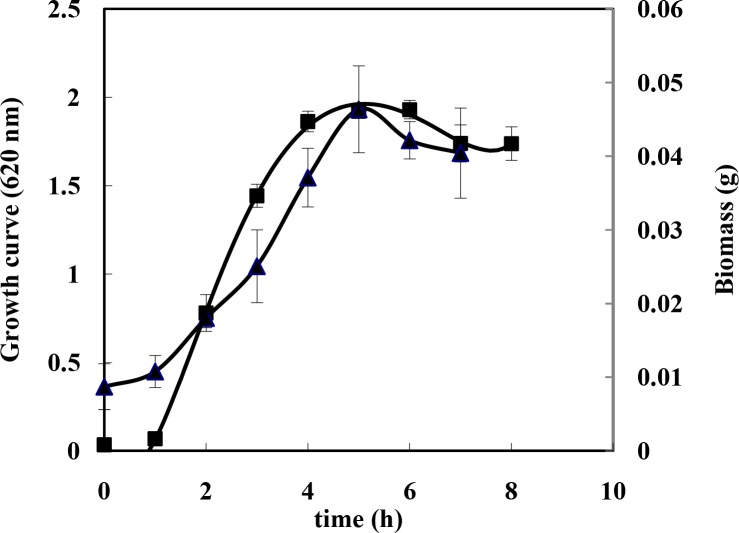
Growth curve (▲) and biomass curves (■) of *B. subtilis*.

**Fig. 4:**
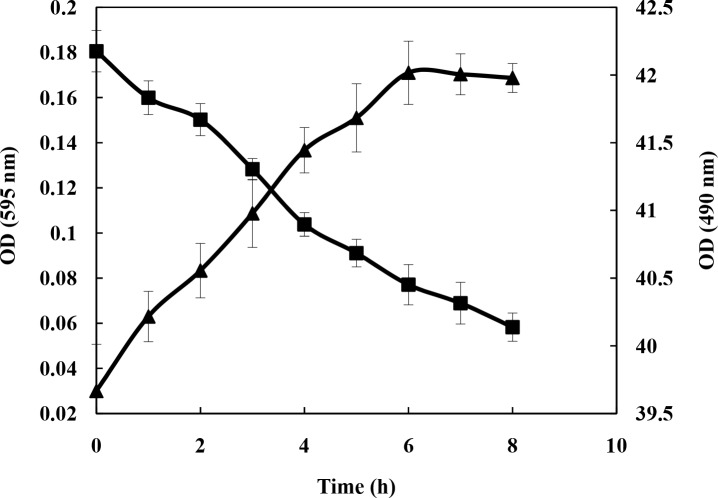
Protein (OD_595_ (◆)) and sugar (OD_490_ (■)) curves of *B. subtilis.*

To evaluate the level of metabolism, protein and sugar concentrations were determined in the culture medium during bacterial growth (Figs. 3–4). The cells were grown in the presence of indole (0.1 g/100 and cane molasses (10 g/L) as carbon source at 28°C. To estimate the protein concentration, Bradford method was used for which a small aliquot of culture medium was assayed in an hour for protein. The binding of dye to protein causes maximum absorption in 595 nm. Moreover, a small aliquot of culture medium was assayed every hour for sugar using phenol sulfuric acid method by checking the readings at *A*_490_. As shown in Figs. 3–4, sugar and protein concentrations decreased and increased, respectively, per hour until maximum cell growth (as noted by changes in *A*_620_ and weight of biomass). The cells in the stationary phase were used for next experiments.

### Monitoring tryptophan production by HPLC

**Fig. 5:**
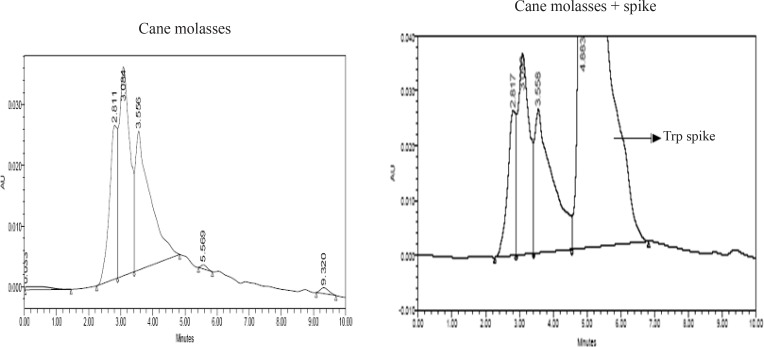
Cane molasses was analyzed in term of the presence of tryptophan using HPLC and its spike. AU means Absorbance Unit in 220 nm.

Tryptophan production by *B. subtilis* was evaluated in the presence of Iranian cane molasses using HPLC (Fig. 5). The results indicate the absence of tryptophan in molasses and in the medium after cell growth. Due to the high concentration of different substances in the samples, discrepancies were observed during the run following injection of samples to the HPLC column. For this reason, TLC was used to follow tryptophan production in next experiments.

### Effect of precursors on tryptophan production monitored by TLC

**Fig. 6:**
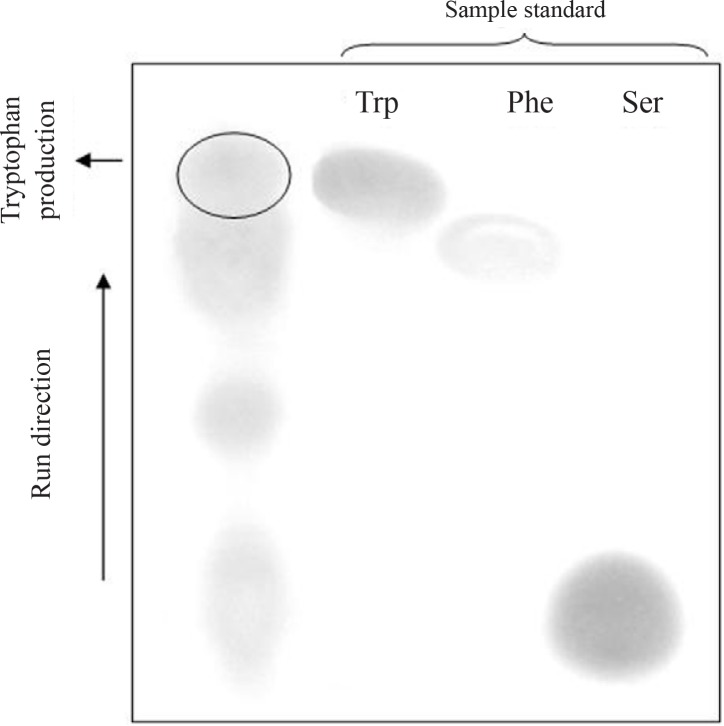
TLC pattern of amino acids in culture medium under different conditions.

Tryptophan production was monitored using TLC in the presence and absence of its two precursors (indole and serine) in medium containing either sucrose or molasses as serine on cell growth. These precursors were added 6 h after cultures were set up using cells from the stationary. After staining by Ninhydrin, the tryptophan spots (purple-brown) in TLC pattern are circular, with a diameter of 2.5-3.0 mm (Retention factor, R_f_ =0.54) and are distinguishable from the spot corresponding to phenylalanine (purple) which is in proximity of the tryptophan spot (Fig. 6). In order to investigate tryptophan spot, indole and PLP standard (as negative control) were also used. The results showed that ninhydrin reagent is linked only to the primary type amine of tryptophan and the scanning amine in indole as well as the ternary amine of PLP is not detected using this method (data not shown). Phe, Trp and Ser are standard samples.

**Fig. 7:**
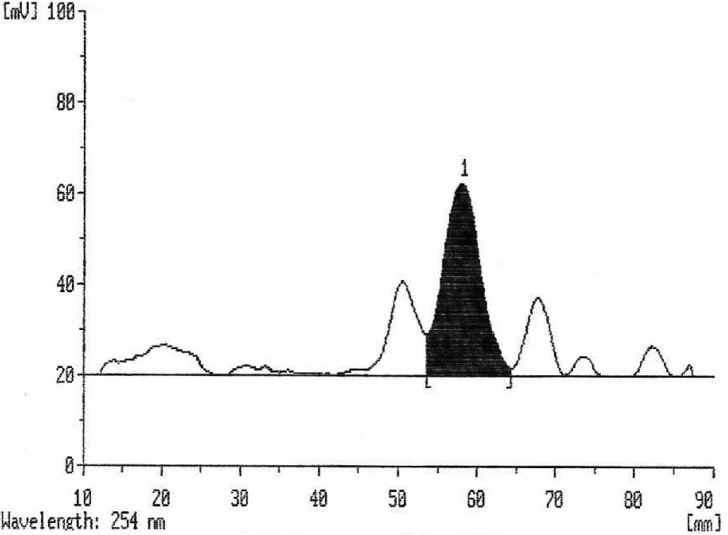
The resulting TLC scan and tryptophan peak area as an example.

Scanning of TLC chromatograms and calculation of the peak areas indicated the amount of tryptophan produced in the samples using standard curve. Fig. 7 shows a typical TLC scan and tryptophan peak area as an example.

**Fig. 8:**
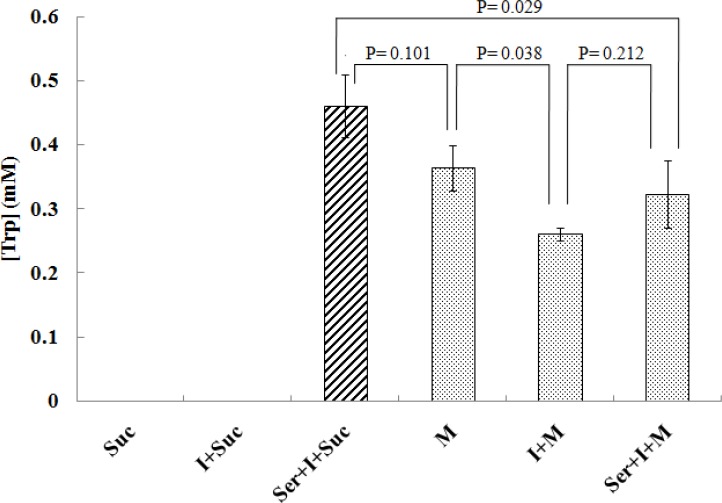
Averages of peak areas resulting from three repeated experiments.

Tryptophan production by *B. subtilis* in the presence and absence of its precursors (0.1% indole, I; 0.2% serine, Ser as well as 1% sucrose, Suc or 10 g/L molasses, M are shown in Fig. 8. The culture medium components and their concentrations have been mentioned in Materials & Methods as sample 1-6. The significance of differences between each of the two given conditions has been expressed as P-value calculated using SPSS software.

## DISCUSSION

Tryptophan is an amino acid necessary for growth in infants and for nitrogen balance in adults and is an essential amino acid. As cane molasses are cheaper than other carbon sources, and are mainly composed of sucrose and glucose, it is a suitable candidate for bacterial growth. The optimum cane molasses concentration for *B. subtilis* growth is 10 g/L which corresponds to 62.4 g/L sugar concentration in culture medium (Fig. 2). As the obtained data indicates, the biomass started to decrease when molasses concentrations were increased to more than 10 g/mL. This observation is probably related to growth inhibitory effect of sugar component of the molasses, a decrease in hydrolysis rate of sugar or osmotic effect of sugar high concentrations (32). The optimum molasses concentration (10 g/L) was used in the following experiments.

Increase in protein concentration and decrease in sugar content of medium, during the bacterial growth, demonstrate active metabolism of *B. subtilis* (Figs. 3, 4) indicative of sugar consumption and protein production by the bacteria from the molasses (the only carbon source in medium). This observation supports the notion that the bacteria produce metabolites (like tryptophan) optimally during the stationary phase and were therefore used in the subsequent experiments. On the other hand, the data show that *B. subtilis* can grow and synthesize proteins without the addition of precursors of tryptophan synthesis (indole and serine) to medium. This might be due to presence of enough tryptophan for growth in yeast extract (0.22 ± 0.05 mM) and peptone (0.14 ± 0.03 mM) as components of culture medium, because *B. subtilis* has been reported to be a tryptophan auxotroph (27).

The lack of a distinguishable tryptophan peak in comparison with spike peak, in HPLC graph confirms the absence of tryptophan in cane molasses (Fig. 5). However, the presence of cane molasses in cell culture generated interesting results (Fig. 8): Although sucrose is a suitable carbon and energy source for cell growth (33), it is not enough for tryptophan production. The addition of indole to sucrose does not seem to be effective either. However, precursors (indole and serine) are required for a prominent increase in tryptophan production (Fig. 8). The results from the use of molasses also point to the fact that this carbon source can be used for tryptophan production albeit less than when it is replaced by serine, indole and sucrose. But this decrease is insignificant (P = 0.101) (Fig. 8). By adding indole to molasses, a significant decrease (p = 0.038) was observed in tryptophan production, due to inhibitory effect of higher concentrations of indole on the second phase of action mechanism of tryptophan synthase (34). No significant change in tryptophan production was observed by adding serine to molasses (Fig. 8).

Results from our previous investigation on tryptophan production by *E. coli* (22) indicate that in this bacterium, the addition of molasses as sole carbon source is sufficient to increase tryptophan production when compared to the use of serine, indole and sucrose. It is postulated that this discrepancy is due to the presence of both tryptophan synthase (TSase; EC 4.2.1.20) and tryptophanase (TPase; EC 4.1.99.1) enzymes in *E. coli* involved in tryptophan production while *B. subtilis* is reported to have tryptophan synthetase only (35).

## CONCLUSIONS

In summary, this report evaluates the potential of cane molasses as an economical source for tryptophan production by *B. subtilis*, hence eliminating the requirement for additional serine and indole as precursors. This study also validated previous investigations reporting the inhibitory effects of indole on tryptophan production.
